# TIR-Domain-Containing Adapter-Inducing Interferon-β (TRIF)-Dependent Antiviral Responses Protect Mice against Ross River Virus Disease

**DOI:** 10.1128/mbio.03363-21

**Published:** 2022-01-04

**Authors:** Xiang Liu, Adam Taylor, Yee Suan Poo, Wern Hann Ng, Lara J. Herrero, Patrick Chun Hean Tang, Ali Zaid, Suresh Mahalingam

**Affiliations:** a Institute for Glycomics, Griffith University, Gold Coast, Queensland, Australia; b Menzies Health Institute Queensland, Griffith University, Gold Coast, Queensland, Australia; c Global Virus Network (GVN) Centre of Excellence in Arboviruses, Griffith University, Gold Coast, Queensland, Australia; d School of Medical Sciences, Griffith University, Gold Coast, Queensland, Australia; University of Colorado School of Medicine

**Keywords:** Ross River virus, alphavirus, innate immunity

## Abstract

Ross River virus (RRV) is the major mosquito-borne virus in the South Pacific region. RRV infections are characterized by arthritic symptoms, which can last from several weeks to months. Type I interferon (IFN), the primary antiviral innate immune response, is able to modulate adaptive immune responses. The relationship between the protective role of type I IFN and the induction of signaling proteins that drive RRV disease pathogenesis remains poorly understood. In the present study, the role of TIR-domain-containing adapter-inducing interferon-β (TRIF), an essential signaling adaptor protein downstream of Toll-like receptor (TLR) 3, a key single-stranded RNA (ssRNA)-sensing receptor, was investigated. We found that TRIF^−/−^ mice were highly susceptible to RRV infection, with severe disease, high viremia, and a low type I IFN response early during disease development, which suggests the TLR3-TRIF axis may engage early in response to RRV infection. The number and the activation level of CD4^+^ T cells, CD8^+^ T cells, and NK cells were reduced in TRIF^−/−^ mice compared to those in infected wild-type (WT) mice. In addition, the number of germinal center B cells was lower in TRIF^−/−^ mice than WT mice following RRV infection, with lower titers of IgG antibodies detected in infected TRIF^−/−^ mice compared to WT. Interestingly, the requirement for TRIF to promote immunoglobulin class switch recombination was at the level of the local immune microenvironment rather than B cells themselves. The slower resolution of RRV disease in TRIF^−/−^ mice was associated with persistence of the RRV genome in muscle tissue and a continuing IFN response.

## INTRODUCTION

Ross River virus (RRV), a member of the genus *Alphavirus* and the *Togaviridae* family, is the most widely spread arbovirus in Australia and can be found throughout the South Pacific region (1, 2). RRV infection in humans was first recorded in 1928 in New South Wales, Australia (3). Several sporadic epidemics of RRV have occurred in recent decades, the largest of which occurred from 1979 to 1980 and involved several countries, including Papua New Guinea, Fiji, Samoa, New Caledonia, and the Cook Islands. In Fiji alone, more than 500,000 people were infected, and 10% of those developed full clinical signs of RRV disease (4, 5). Each year in Australia, approximately 5,000 clinical cases of RRV disease (RRVD) are reported (6, 7). In 2015, RRVD cases reached a 23-year high with 9,542 cases reported (8). In addition to fever and lethargy, patients infected with RRV develop polyarthritis, myalgia, and sometimes rash (9). A subset of patients develop chronic arthralgia and debilitating pain that can last for months to years, resulting in a significant health, economic, and social burden. The annual cost of RRVD in Australia, including testing, treatment, and lost earnings, is estimated to be around AU$20 million (7). There are currently no specific treatments or vaccines available for RRV infection, and analgesics and nonsteroidal anti-inflammatory drugs (NSAIDs) are the principal treatment provided to symptomatic patients (9).

Type I interferons (IFNs) are the host’s first line of defense against viral infections. They also play important roles in the modulation of adaptive immune responses and immune cell differentiation (10). Type I IFN production can be stimulated by a number of different signaling pathways depending on the viral pathogen. The RIG-like helicase (RLH) family and Toll-like receptor (TLR) family induction pathways are essential in the detection of alphavirus-generated pathogen-associated molecular pattern (PAMP) RNA. During alphavirus infection, the IFN-β promoter stimulator 1 (IPS-1)-dependent retinoic acid-inducible gene 1 (RIG-I) pathway, myeloid differentiation primary response gene 88 (Myd88)-dependent TLR7, pathway and TIR-domain-containing adapter-inducing IFN-β (TRIF)-dependent TLR3 pathway may independently contribute to the expression of type I IFN (11–21). TLR3 is a key cytoplasmic sensor that can recognize foreign double-stranded RNA (dsRNA) and has been shown to sense RNA from influenza virus, chikungunya virus (CHIKV), Japanese encephalitis virus (JEV), hepatitis C virus (HCV), hepatitis A virus (HAV), and dengue virus (DENV) (21–26). TRIF is the adaptor downstream of the TLR3 signaling cascade and interacts with TBK1 (TANK [TRAF (tumor necrosis factor receptor-associated factor) family member-associated NF-kappa-B activator] binding kinase 1) and IKKε/IKKi (IκB [inhibitor of NF-κB (nuclear factor κB)] kinase) (27, 28). TRIF contributes to protection of the host from H5N1 influenza virus and herpes simplex virus 1 (HSV-1) infections (29, 30). In addition, TRIF was identified to be a proteolysis target by invading HAV and HCV, suggesting TRIF plays a crucial role in the induction of type I IFN following infection by RNA viruses (25, 31). In a mouse model of CHIKV infection, TRIF^−/−^ mice were more susceptible to CHIKV-induced inflammation, concomitant with impaired type I IFN expression (20). However, more detail on the interplay between the adaptive immune responses and TRIF/type I IFN axis during alphavirus infection is needed to better understand RRVD pathogenesis. In this study, the protective roles of TRIF and type I IFN were investigated using a mouse model of RRVD. Here, we investigate the role of TRIF in the kinetics of neutralizing antibody production, antiviral and proinflammatory cytokine production, tissue leukocyte infiltration, and lymphocyte differentiation to determine the requirement of TRIF-induced signaling in RRVD.

## RESULTS

### TRIF^–/–^ mice develop severe disease during RRV infection.

**FIG 1:**
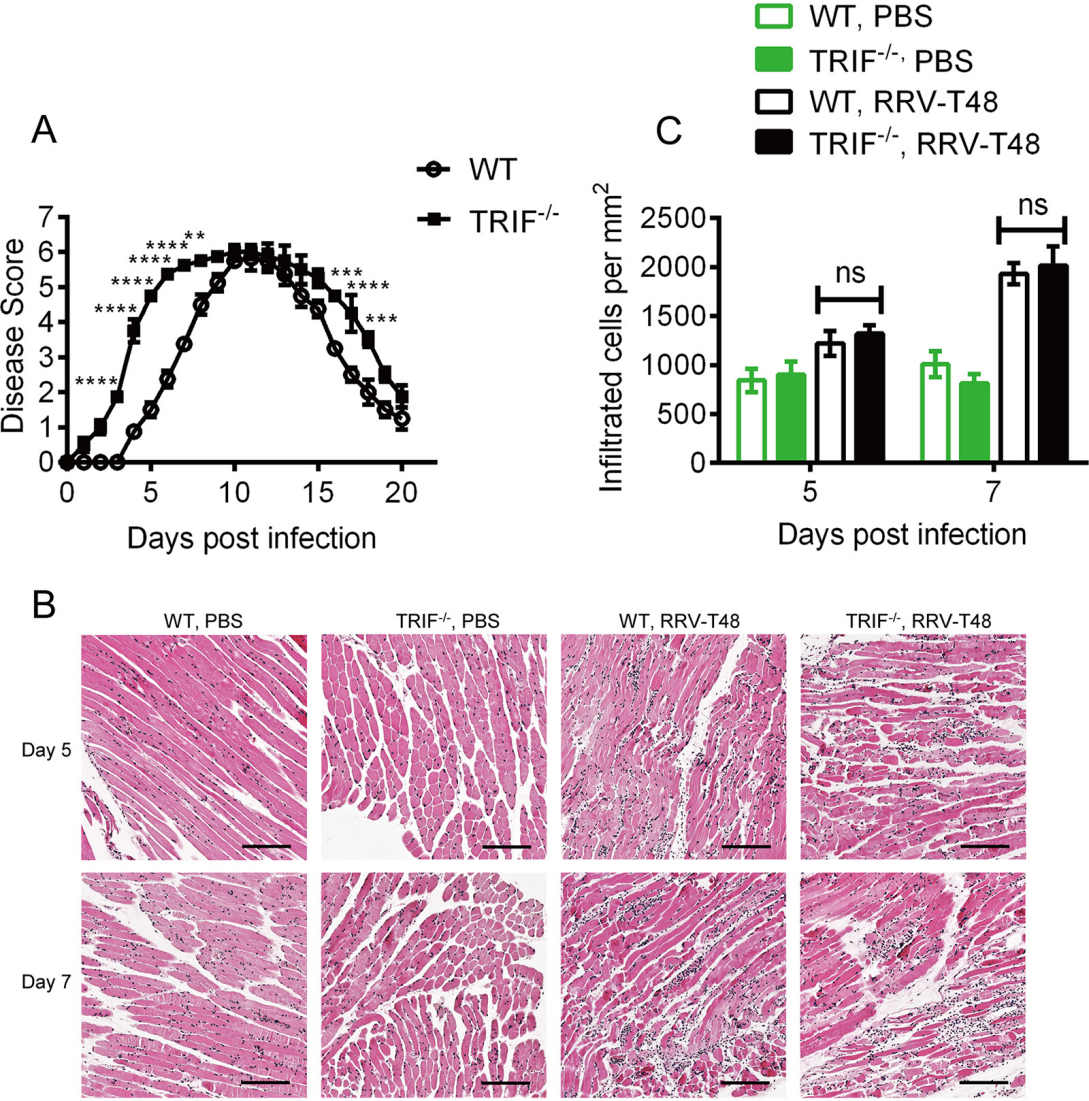
RRV disease and inflammatory cell infiltrates in RRV-infected WT and TRIF^−/−^ mice. First 20-day-old C57BL/6 wild-type (WT) or TRIF^−/−^ mice were injected subcutaneously with 10^4^ PFU RRV or mock-infected with PBS. (A) Mouse clinical scores were monitored daily. Mice were scored according to hind limb strength and onset of hind limb dysfunction. Mock-infected mice showed no disease signs for the duration of the experiment. All values represent the mean ± standard error of the mean (SEM) of 5 mice per group of duplicate experiments. (**, *P *< 0.01; ***, *P* < 0.001; ****, *P* < 0.0001 using repeated-measure two-way ANOVA with Bonferroni posttest). (B) H&E staining of longitudinal sections of quadriceps from mice infected with RRV T48 at days 5 and 7 p.i. Images are shown as ×10 magnification with thresholded nuclei shown in dark blue. Scale bar = 100 μm. Threshold signal was used for automated quantitation of nuclei and indicates tissue-infiltrating cells. (C) The statistical analysis of cell infiltrates was quantified using ImageScope. All values represent the mean ± SEM of 5 mice per group of duplicated experiments (statistical analysis performed using two-way ANOVA with Bonferroni posttest).

To compare the severity of RRV disease between TRIF knockout (TRIF^−/−^) and wild-type (WT) mice, groups of mice were infected subcutaneously with 10^4^ PFU of RRV. Disease signs were monitored daily until day 20 postinfection (p.i.). Compared to WT mice, TRIF^−/−^ mice infected with RRV developed rapid hind limb weakness and loss of gripping ability (Fig. 1A). At days 10 to 12 p.i., which correspond with peak disease in TRIF^−/−^- and WT-infected mice, both groups displayed similar disease scores. The results suggest that TRIF may play a protective role in the development of RRV disease.

The rapid onset of disease in TRIF^−/−^-infected mice suggests the initiation of an early inflammatory response in target tissues. To examine whether early disease onset in TRIF^−/−^ mice was associated with increased inflammatory cell infiltration in quadricep muscle, TRIF^−/−^ mice and WT mice were infected subcutaneously with 10^4^ PFU of RRV and culled at days 5 and 7 p.i., and quadriceps were harvested. Histological analysis with hematoxylin and eosin (H&E) staining of quadriceps was performed (Fig. 1B), and infiltrating leukocytes were enumerated (Fig. 1C). No difference in the number of immune cell infiltrates in the quadriceps of RRV-infected TRIF^−/−^ mice compared to WT mice was detected at day 5 or 7 p.i. These results indicate that despite rapid onset of severe disease in RRV-infected TRIF^−/−^ mice compared to WT mice, immune cell infiltration in quadriceps at days 5 and 7 p.i. was not enhanced in TRIF^−/−^ mice (Fig. 1).

### Delayed and diminished type I IFN response is associated with increased viremia and spleen virus titers in RRV-infected TRIF^–/–^ mice.

**FIG 2:**
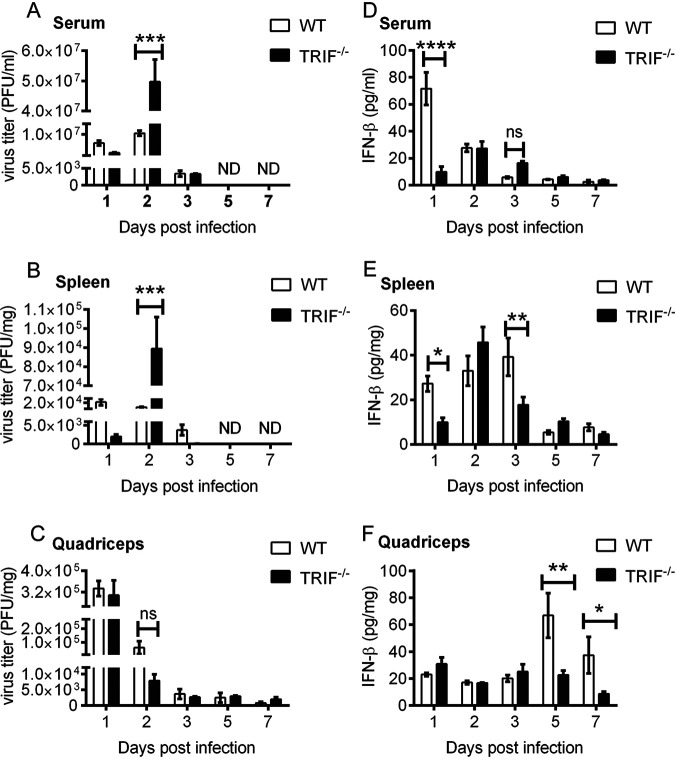
RRV replication kinetics and type I IFN levels in WT and TRIF^−/−^ infected mice. First 20-day-old C57BL/6 WT and TRIF^−/−^ mice were injected subcutaneously with 10^4^ PFU of RRV. (A) Serum, (B) spleens, and (C) quadriceps were collected at days 1, 2, 3, 5, and 7 p.i. Viral titers were determined by plaque assay. (D) Serum, (E) spleens, and (F) quadriceps were collected at days 1, 2, 3, 5, and 7 p.i. Type I IFN levels were measured by ELISA. Values are shown as the mean (5 mice per group) ± SEM of duplicated experiments. ND, not detected (ns, not significant; *, *P* < 0.05; **, *P* < 0.01; ***, *P* < 0.001, ****, *P* < 0.001 using two-way ANOVA with Bonferroni posttest).

To determine whether severe disease in TRIF^−/−^ mice was associated with enhanced virus replication, virus titer was determined in the serum, spleen, and quadricep tissue of infected mice. TRIF^−/−^ and WT mice were infected subcutaneously with 10^4^ PFU of RRV and sacrificed at days 1, 2, 3, 5, and 7 p.i. Serum and tissues were collected for plaque assay analysis. Compared to WT mice, RRV was detected at significantly higher titers in the serum (∼4.7-fold) and spleen (∼9.1-fold) of TRIF^−/−^ mice at day 2 p.i. (Fig. 2A and B). At days 1 and 3 p.i., virus titers were higher in the serum and spleen of WT mice compared to TRIF^−/−^ mice (Fig. 2A and B), although the differences were not statistically significant. Virus titers in the serum and spleen of all infected mice were below the limit of detection by plaque assay at days 5 and 7 p.i. There were also no significant differences in viral titers recovered from the quadriceps of WT and TRIF^−/−^ mice (Fig. 2C). These results suggest that severe rapid onset of disease in TRIF^−/−^ mice correlated with high viremia and viral load in serum and spleen at day 2 p.i. and that TRIF may contribute to controlling systemic replication of RRV.

To determine whether TRIF controls RRV replication through type I IFN responses, the levels of type I IFN in TRIF^−/−^ infected mice were determined in the spleen, serum, and quadriceps by enzyme-linked immunosorbent assay (ELISA). At day 1 p.i., the mean levels of type I IFN in the serum of TRIF^−/−^ mice were approximately 10 pg/mL, ∼7-fold lower than that of WT mice (Fig. 2D). Serum IFN levels increased to approximately 30 pg/mL in TRIF^−/−^ mice at day 2 p.i., a similar concentration to that seen in WT mice. At day 3 p.i., type I IFN levels in both TRIF^−/−^ and WT mice began to decrease to approximately 18 and 8 pg/mL, respectively.

Splenic levels of type I IFN were again significantly reduced in infected TRIF^−/−^ mice on day 1 p.i. compared to WT mice (Fig. 2E). Mean levels of type I IFN peaked in the spleen of infected TRIF^−/−^ mice on day 2 p.i. at approximately 46 pg/mL before decreasing. However, significant levels of type I IFN were maintained in the spleen of infected WT mice until day 3 p.i.

In quadriceps, no significant differences in the levels of type I IFN were detected between infected TRIF^−/−^ and WT mice at days 1, 2, and 3 p.i. (Fig. 2F). However, a sharp increase in the levels of IFN in the quadriceps of infected WT mice was detected at day 5 p.i. This increase was not observed in TRIF^−/−^ mice. A significant increase in type I IFN levels in the quadriceps of infected WT mice compared to TRIF^−/−^ mice was maintained at day 7 p.i. (Fig. 2F).

The low levels of type I IFN in the serum and the spleen of TRIF^−/−^ mice at day 1 p.i. may be responsible for the high viremia and spleen viral load in TRIF^−/−^ mice at day 2 p.i. The results also suggest a crucial role for TRIF in the early stages of type I IFN induction during RRV infection. However, the increase in type I IFN levels in the spleens of TRIF^−/−^ mice at day 2 p.i. suggests a TRIF-independent mechanism for complementary type I IFN induction. The increase in type I IFN levels in the quadriceps of infected WT mice at days 5 and 7 p.i. likely reflects the continued replication of RRV in these tissues at these time points. Although not significant, virus titer in the quadriceps of infected TRIF^−/−^ mice increased compared to that of WT mice at day 7 p.i., possibly owing to limited IFN production in these tissues.

### CCL5 and IL-15 mRNA expression are upregulated in RRV-infected TRIF^–/–^ mice.

**FIG 3:**
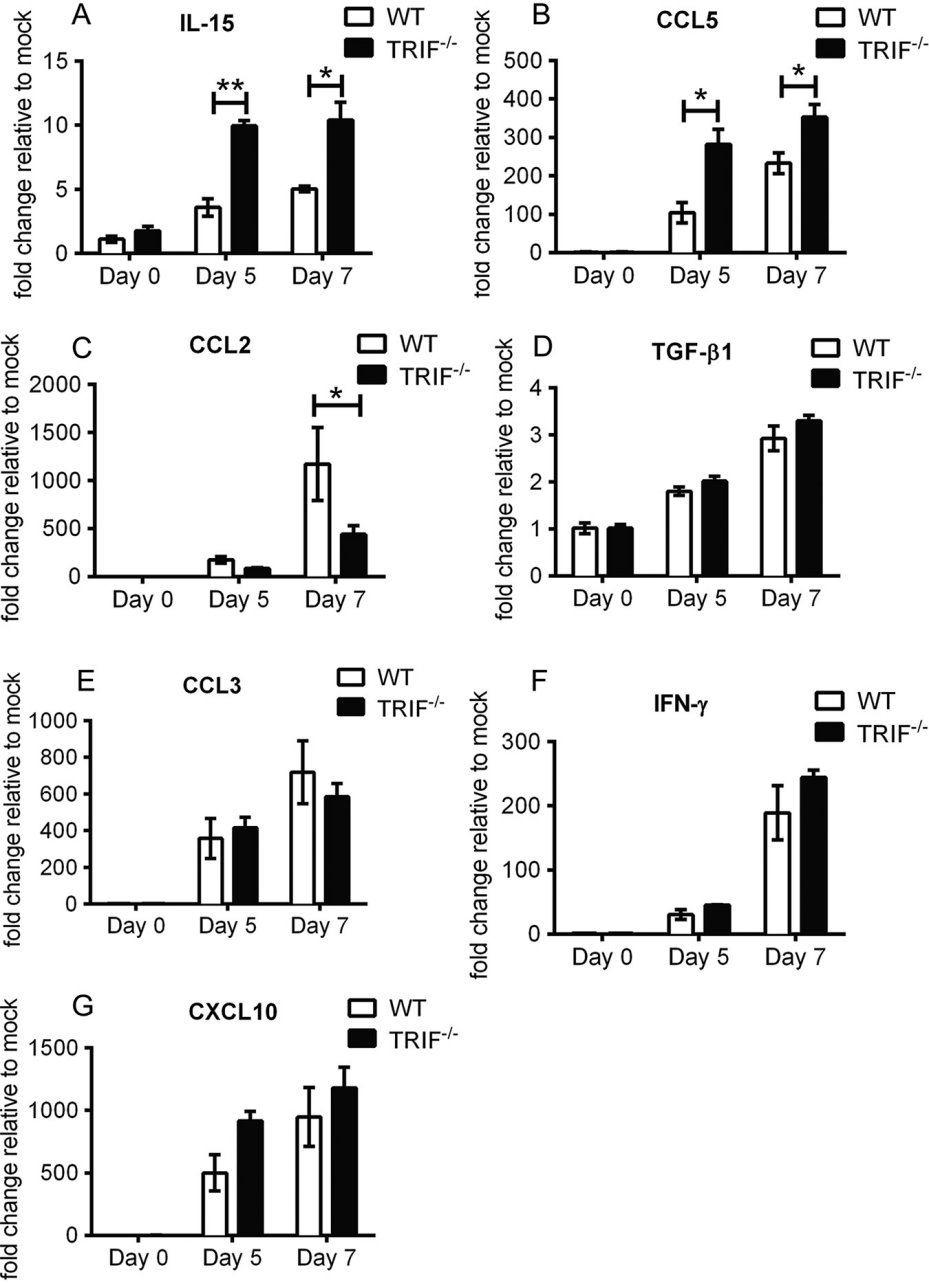
Cytokine profile in WT and TRIF^−/−^ RRV-infected mice. First, 20-day-old C57BL/6 WT and TRIF^−/−^ mice were injected subcutaneously with 10^4^ PFU of RRV. (A to G) Quadriceps were processed for qRT-PCR assay for IL-15 (A), CCL5 (RANTES) (B), CCL2 (MCP-1) (C), TGF-β1 (D), CCL3 (MIP-1α) (E), IFN-γ (F), and CXCL10 (IP-10) (G) at days 5 and 7 p.i. Values are shown as the mean (5 mice per group) ± SEM of duplicated experiments. (*, *P* < 0.05; **, *P* < 0.01 using two-way ANOVA with Bonferroni posttest).

To understand how the TRIF/type I IFN axis drives antiviral and proinflammatory cytokine production during RRV disease, we assessed mRNA expression levels of IFN-γ, transforming growth factor β1 (TGF-β1), interleukin-15 (IL-15), C-C motif ligand 2 (CCL2), CCL3, CCL5, and C-X-C motif ligand 10 (CXCL10) in the quadriceps muscle of TRIF^−/−^ and WT mice at days 5 and 7 p.i. These time points correspond to the onset and early disease phase in RRV-infected TRIF^−/−^ mice, respectively. These cytokines and chemokines are known to be important mediators of innate and adaptive immune responses during RRV infection (32–36). The mRNA levels of IL-15 and CCL5 in TRIF^−/−^ mice were both significantly higher than in WT mice at days 5 and 7 p.i. (Fig. 3A and B). mRNA levels of CCL2, a key driver of inflammation in RRV disease, were higher in WT mice than in TRIF^−/−^ mice at day 7 p.i. (Fig. 3C). No significant differences were observed in the mRNA levels for TGF-β1, CCL3, IFN-γ, and CXCL10 between TRIF^−/−^ and WT mice (Fig. 3D to G). These data suggest that TRIF-dependent signaling may play a role in regulating IL-15, CCL5, and CCL2 mRNA expression during the development of RRV disease, potentially resulting in early onset of severe disease signs in infected TRIF^−/−^ mice.

### Reduced infiltration and impaired activation of T cells and NK cells in RRV-infected TRIF^–/–^ mice.

**FIG 4:**
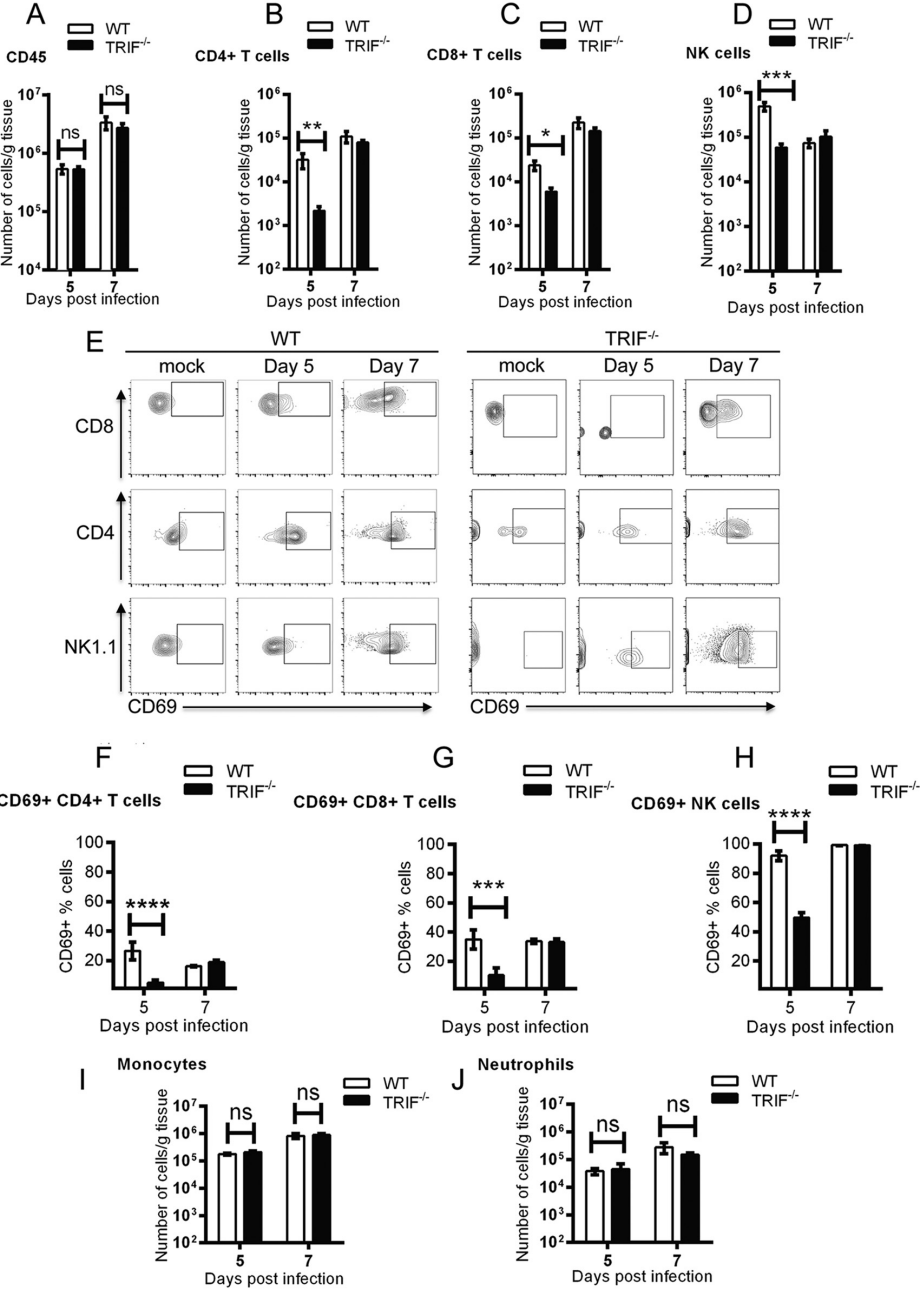
Immune cell responses in WT and TRIF^−/−^ RRV-infected mice. First, 20-day-old C57BL/6 WT and TRIF^−/−^ mice were injected subcutaneously with 10^4^ PFU of RRV. Quadricep muscle was processed for flow cytometry analysis at days 5 and 7 p.i. (A to D) The numbers of (A) CD45^+^ leukocytes, (B) CD4^+^, (C) CD8^+^ T cells, and (D) NK1.1^+^ NK cells were analyzed. (E) The gating strategy. (F to J) The percentages of (F) CD69^+^ CD4^+^, (G) CD69^+^ CD8^+^ T cell, and (H) CD69^+^ NK1.1^+^ NK cells and the numbers of (I) CD11b^hi^Ly6^Chi^ monocytes and (J) Ly6G^+^ neutrophils were analyzed. Values are shown as the mean (6 mice per group) ± SEM of duplicated experiments. (ns, not significant; *, *P* < 0.05; **, *P* < 0.01; ***, *P* < 0.001; ****, *P* < 0.0001 using two-way ANOVA with Bonferroni posttest).

Because a lack of TRIF-dependent signaling led to an increase in IL-15 and CCL5, both of which regulate T cell and NK cell maintenance and proliferation (37, 38), we hypothesized that T cell and NK cell responses were impaired in TRIF^−/−^ mice. Quadriceps were collected at days 5 and 7 p.i. and processed for flow cytometry. As shown in Fig. 4A, there was no difference in the overall number of CD45^+^ leukocytes between infected WT mice and TRIF^−/−^ mice. However, the numbers of CD4^+^ T cells, CD8^+^ T cells, and NK cells at day 5 p.i. were significantly lower in TRIF^−/−^ mice compared to WT mice (Fig. 4B to D). Interestingly, when assessing lymphocyte activation through expression of CD69, a transmembrane C-type lectin associated with effector T cell phenotype and NK cell activation, the percentages of CD69^+^ CD4^+^ T cells, CD8^+^ T cells, and NK cells in TRIF^−/−^ mice at day 5 p.i. were significantly lower in TRIF^−/−^ mice (4.9 ± 1.8%, 10.4 ± 5.0%, and 49.6 ± 3.4%, respectively) compared to WT mice, (26.5 ± 6.1%, 34.9 ± 6.6%, and 91.9 ± 3.3%, respectively) (Fig. 4F to H). In contrast, at day 7 p.i., the numbers and activation levels of CD8^+^ CD4^+^ T cells and NK cells in TRIF^−/−^ mice were similar to those of WT mice. There were no significant differences in the number of infiltrating myeloid cells, including CD11b^hi^Ly6C^hi^ inflammatory monocytes or Ly6G^+^CD11b^hi^Ly6C^int^SSC^hi^ neutrophils, between TRIF^−/−^ mice and WT mice at days 5 and 7 p.i. (Fig. 4I and J). As functional type I IFN signaling is essential to generate effective leukocyte responses, the delayed expansion and activation of CD8^+^ T cells, CD4^+^ T cells, and NK cells at day 5 p.i. in TRIF^−/−^ infected mice may be a consequence of curtailed type I IFN responses at early time points postinfection.

### Impaired immunoglobulin class switch recombination in RRV-infected TRIF^–/–^ mice.

**FIG 5:**
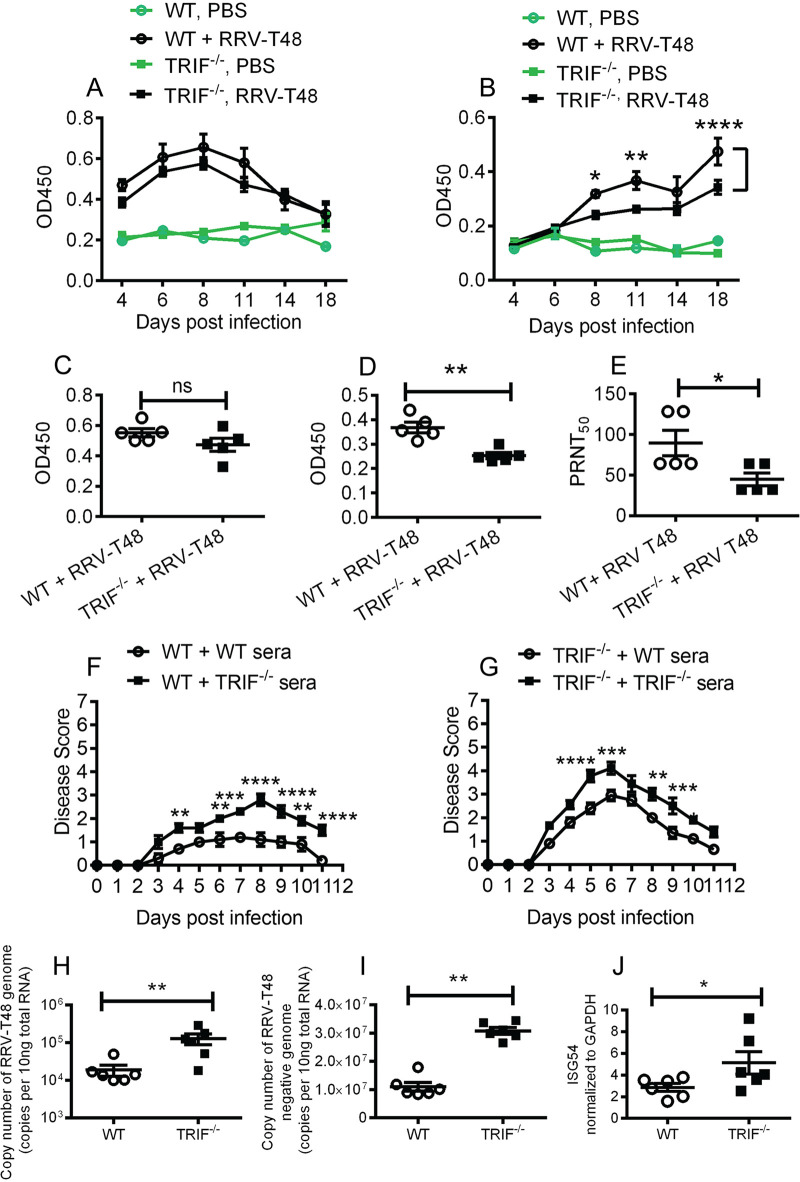
Antibody responses in TRIF^−/−^ mice infected with RRV. C57BL/6 WT and TRIF^−/−^ mice were injected subcutaneously with 10^4^ PFU of RRV or mock-infected with PBS. (A and B) Sera were collected on days 4, 6, 8, 11, 14, and 18 p.i. and processed for (A) IgM and (B) IgG ELISA. Values are shown as the mean (5 mice per group) ± SEM of duplicated experiments (*, *P* < 0.05; **, *P* < 0.01; ****, *P* < 0.0001 using two-way ANOVA with Bonferroni posttest). (C and D) Sera were collected on day 10 p.i. and processed for (C) IgM and (D) IgG ELISA. Values are shown as the mean (5 mice per group) ± SEM of duplicated experiments. (**, *P* < 0.01 using Student’s *t* test). (E) The sera collected at day 10 p.i. were also used for RRV neutralizing antibody analysis using the PRNT_50_ assay. Neutralizing antibody titers are expressed as the inverse of the dilution where 50% of the virus present was neutralized (PRNT_50_) for a given sample. Values are shown as the mean (5 mice per group) ± SEM of duplicated experiments. (*, *P < *0.05 using Student’s *t* test). The 20-day-old C57BL/6 WT and TRIF^−/−^ mice were injected intraperitoneally with 50 μL of heat-inactivated sera from RRV-infected C57BL/6 WT or TRIF^−/−^ mice collected at day 10 p.i. Mice were then injected subcutaneously with 10^4^ PFU of RRV 1 h after serum transfer. (F and G) Mouse disease scores were monitored daily. All values represent the mean ± SEM of 5 mice per group of duplicate experiments. (*, *P* < 0.05; **, *P < *0.01; ***, *P* < 0.001; ****, *P* < 0.0001 using repeated-measure two-way ANOVA with Bonferroni posttest). Then, 20-day-old C57BL/6 WT and TRIF^−/−^ mice were injected subcutaneously with 10^4^ PFU of RRV. (H to I) Quadriceps were processed by qRT-PCR assay for viral genome (H), viral negative-strand genome (I), and ISG54 (J) at day 18 p.i. Values are shown as the mean (6 mice per group) ± SEM of duplicated experiments. (*, *P* < 0.05; **, *P* < 0.01 using Student’s *t* test).

Our observation that TRIF^−/−^ mice do not recover from disease until later time points (e.g., days 18 to 20 p.i.) suggests that in addition to cellular responses, antibody responses may be impaired in the absence of TRIF signaling. Robust neutralizing antibody responses are crucial for viral clearance and disease recovery; therefore, we asked whether RRV-specific antibody titers were impaired in TRIF^−/−^ mice. Blood was collected at days 4, 6, 8, 11, 14, and 18 p.i., and anti-RRV IgM (Fig. 5A) and IgG (Fig. 5B) titers were measured by ELISA. IgM titers were comparable between WT and TRIF^−/−^ mice from 4 dpi to 18 dpi, while IgG titers in TRIF^−/−^ mice were significantly lower than in WT mice at days 8, 11, and 18 p.i.

To confirm that antibodies generated in WT mice following infection were a determining factor in abating disease severity, we performed an adoptive serum transfer experiment using serum from WT and TRIF^−/−^ mice that had been infected 10 days previously with 10^4^ PFU RRV. This 10-day serum showed the same pattern of antibody levels that had been observed at days 8, 11, and 18 p.i.; that is, anti-RRV IgM titers were not significantly different (Fig. 5C), while IgG titers in TRIF^−/−^ mice were significantly lower than in WT mice (Fig. 5D). The neutralizing titer of the antibodies were measured by a 50% plaque reduction/neutralization titer (PRNT_50_) assay using RRV-T48 (10^3^ PFU/mL) (Fig. 5E). The titer of the neutralizing antibodies from RRV-infected WT mice was approximately 90, whereas the titer of the sera from TRIF^−/−^ mice was approximately 45. The sera at day 10 p.i. were transferred to WT or TRIF^−/−^ mice prior to RRV infection. Over the course of infection, WT mice that received sera from TRIF^−/−^ mice developed more severe disease compared to mice that received sera from WT mice, suggesting that IgG antibody titers generated in RRV-infected TRIF^−/−^ mice were not sufficient to protect mice from RRVD (Fig. 5F). Similarly, TRIF^−/−^ mice that received the sera from TRIF^−/−^ mice also developed more severe disease compared to TRIF^−/−^ mice that received sera from WT mice (Fig. 5G). Interestingly, TRIF^−/−^ mice that received sera from TRIF^−/−^ mice presented with more severe disease than WT mice that received sera from TRIF^−/−^ mice. These data indicate that TRIF signaling may be important in humoral antiviral responses during RRV infection.

### Increased viral RNA persistence in TRIF^–/–^ mice after resolution of RRV disease.

As TRIF^−/−^ mice produced lower levels of RRV-specific IgG at later time points, we asked whether the absence of TRIF could facilitate RRV persistence in tissues. Mice were infected with RRV, and the quadriceps muscle was collected at day 18 p.i., and strand-specific reverse transcription-quantitative PCR (qRT-PCR) detection was performed. Copy numbers of RRV-positive (Fig. 5H) and negative-strand (Fig. 5I) RNA in quadriceps were both significantly higher in TRIF^−/−^ mice compared to WT mice at 18 dpi. However, no infectious viral particles were detected by plaque assay in either WT or TRIF^−/−^ mice at this time point. These data suggest that impaired IgG antibody production due to the absence of TRIF may hinder RRV clearance in these tissues. Furthermore, as an indicator of type I IFN activation, higher levels of ISG54 (Fig. 5J) in TRIF^−/−^-infected mice compared to WT mice indicates an ongoing stimulation of type I IFN responses by the remaining RRV RNA. Higher levels of viral RNA and antiviral sensing mechanisms at later time points after infection are thus likely to contribute to the slower resolution of RRV disease in TRIF^−/−^ mice as shown in Fig. 1A.

### Functional TRIF signaling within the immune microenvironment enables B cell differentiation during RRV infection.

To further investigate the possibility of a defect in humoral responses in infected TRIF^−/−^ mice, we initially determined whether germinal center (GC) formation was affected by a lack of TRIF and asked whether GC B cell (commonly defined as B220^+^ GL-7^+^ B cells) development was impaired in TRIF^−/−^ mice. TRIF^−/−^ and WT mice were infected with RRV and at days 5 and 7 p.i., and foot-draining popliteal lymph nodes and spleens were collected for immunofluorescence microscopy and flow cytometry, respectively.

**FIG 6:**
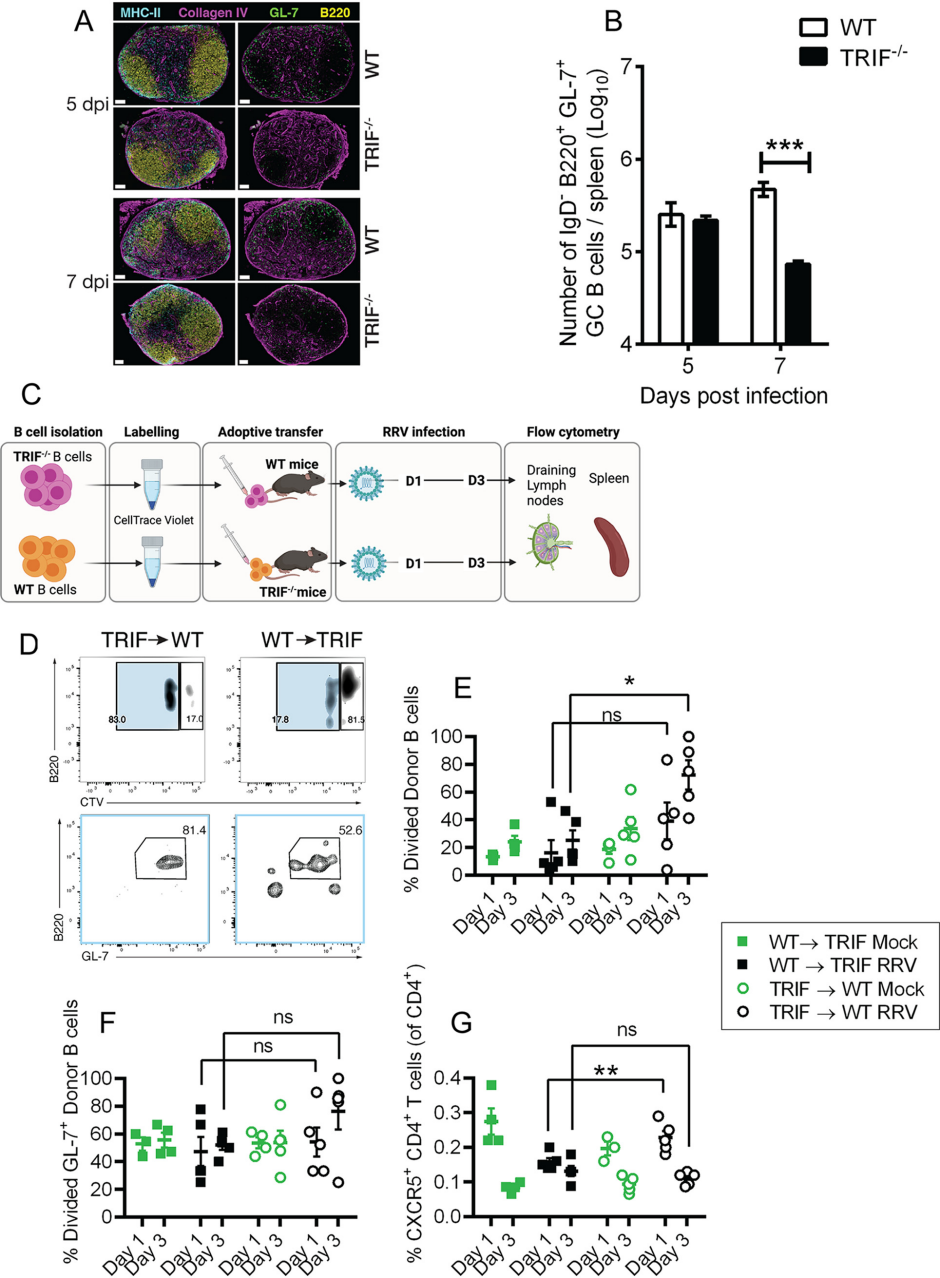
GC B cell activation in TRIF^−/−^ mice infected with RRV. First, 20-day-old C57BL/6 WT and TRIF^−/−^ mice were injected subcutaneously with 10^4^ PFU of RRV or mock-infected with PBS. Spleen and lymph nodes were processed for flow cytometry and immunofluorescence histology analysis at days 5 and 7 postinfection. (A) Confocal immunofluorescence microscopy of GC B cells in foot-draining popliteal lymph nodes. Left panels show merged channels; right panels show collagen IV and GL-7. Scale bar = 100 μm. (B) Flow cytometry analysis and enumeration of GC B cells in the spleens of RRV-infected WT and TRIF^−/−^ mice at 5 and 7 days p.i. Values (log_10_) are shown as the mean (5 mice per group) ± SEM of duplicated experiments. (***, *P* < 0.001 using two-way ANOVA with Bonferroni posttest). (C) Schematic of the adoptive B cell transfer experiment. WT and TRIF^−/−^ B cells were FACS-sorted and labeled using CellTrace violet (CTV) proliferation dye. Then, 2.5 × 10^5^ CTV-labeled B cells were adoptively transferred (intravenously [i.v.]) into mismatched recipients (i.e., WT B cells into TRIF^−/−^ mice and TRIF^−/−^ B cells into WT mice) 1 day before subcutaneous inoculation with RRV or PBS vehicle (mock group). On days 1 and 3 postinfection, pooled draining brachial and axillary lymph nodes (LNbr/ax) and spleens were collected and processed for flow cytometry. (D) Representative gates for CTV^+^ B cells showing adoptively transferred donor B cells that have undergone one round of proliferation (light blue gate, upper panels) in their respective mismatched recipients and subsequent gating for B220^+^GL-7^+^ divided donor B cells (lower panels). B220^+^CTV- cells were gated out prior to the gates shown. Gates show the frequency of parent population. (E) Percentage of divided donor B cells in WT and TRIF^−/−^ mice in the draining lymph nodes at 1 and 3 days postinfection with RRV. (F) Percentage of divided B220^+^GL-7^+^ B cells in WT and TRIF^−/−^ mice in the draining lymph nodes at 1 and 3 days postinfection with RRV. (G) Percentage of CXCR5^+^CD4^+^ follicular helper (T_FH_) T cells in the spleen of WT and TRIF^−/−^ mice at 1 and 3 days postinfection with RRV. Plots show individual mice (*n* = 3 to 5) with mean and SEM. Statistically significant differences shown between WT and TRIF^−/−^ hosts at 1 and 3 days postinfection. Statistically significant differences were determined using a two-way ANOVA with a Sidak test for multiple comparisons. *, *P* < 0.05; **, *P* < 0.01; ns, not significant.

GC B cell staining showed that GL-7^+^ B cells were less abundant in popliteal lymph nodes in TRIF^−/−^ mice compared to WT mice at both 5 and 7 days p.i. (Fig. 6A). In the spleen, the number of GL-7^+^ GC B cells was significantly lower in TRIF^−/−^ mice compared to WT mice at day 7 p.i., but not at day 5 p.i. (Fig. 6B). Together, these results suggest that TRIF^−/−^ mice fail to effectively stimulate GC formation after RRV infection.

These findings do not, however, confirm or refute that class switch recombination was specifically impaired, as the data in Fig. 5A and B suggested. Recent studies showed that class switch recombination can occur independently of, or prior to, GC formation (39). We asked whether defects in early GC formation could account for impaired class switch recombination in TRIF^−/−^ mice. We performed adoptive B cell transfer experiments (Fig. 6C) where B cells from WT and TRIF^−/−^ mice were fluorescence-activated cell sorter (FACS)-sorted and labeled using CellTrace violet (CTV). CTV-labeled donor B cells were adoptively transferred into mismatched recipients 1 day before RRV infection (e.g., TRIF^−/−^ B cells were transferred into WT mice, and WT B cells into TRIF^−/−^ mice). B cell phenotype and localization were examined in their respective hosts in the lymph nodes (FACS) and spleen (immunofluorescence) at days 1 and 3 p.i. (Fig. 6D). TRIF^−/−^ B cells transferred to WT mice were found to proliferate better than WT B cells transferred to TRIF^−/−^ mice (Fig. 6E). In addition, the proportion of divided GL-7^+^ TRIF^−/−^ B cells was higher in WT recipients than that of WT B cells in TRIF^−/−^ recipients (Fig. 6F) at day 3 postinfection. Although these differences were not statistically significant (mean differences, −24.32; 95% confidence interval [CI] of differences, −59.43 to 10.79), these data suggest that functional TRIF signaling within the immune (and potentially stromal) microenvironment could be essential in enabling B cells to undergo early-stage differentiation in the early events of class switch recombination.

Further, the proportion of CXCR5^+^ CD4^+^ follicular helper (T_FH_) T cells in the lymph nodes was significantly higher in WT mice that had received TRIF^−/−^ B cells at day 1 postinfection compared to TRIF^−/−^ mice that received WT B cells (Fig. 6G), corroborating the notion that the local microenvironment—rather than B cells themselves—may require TRIF to potentiate the T_FH_/germinal center (GC) B cell cross talk, leading to class switch recombination.

**FIG 7:**
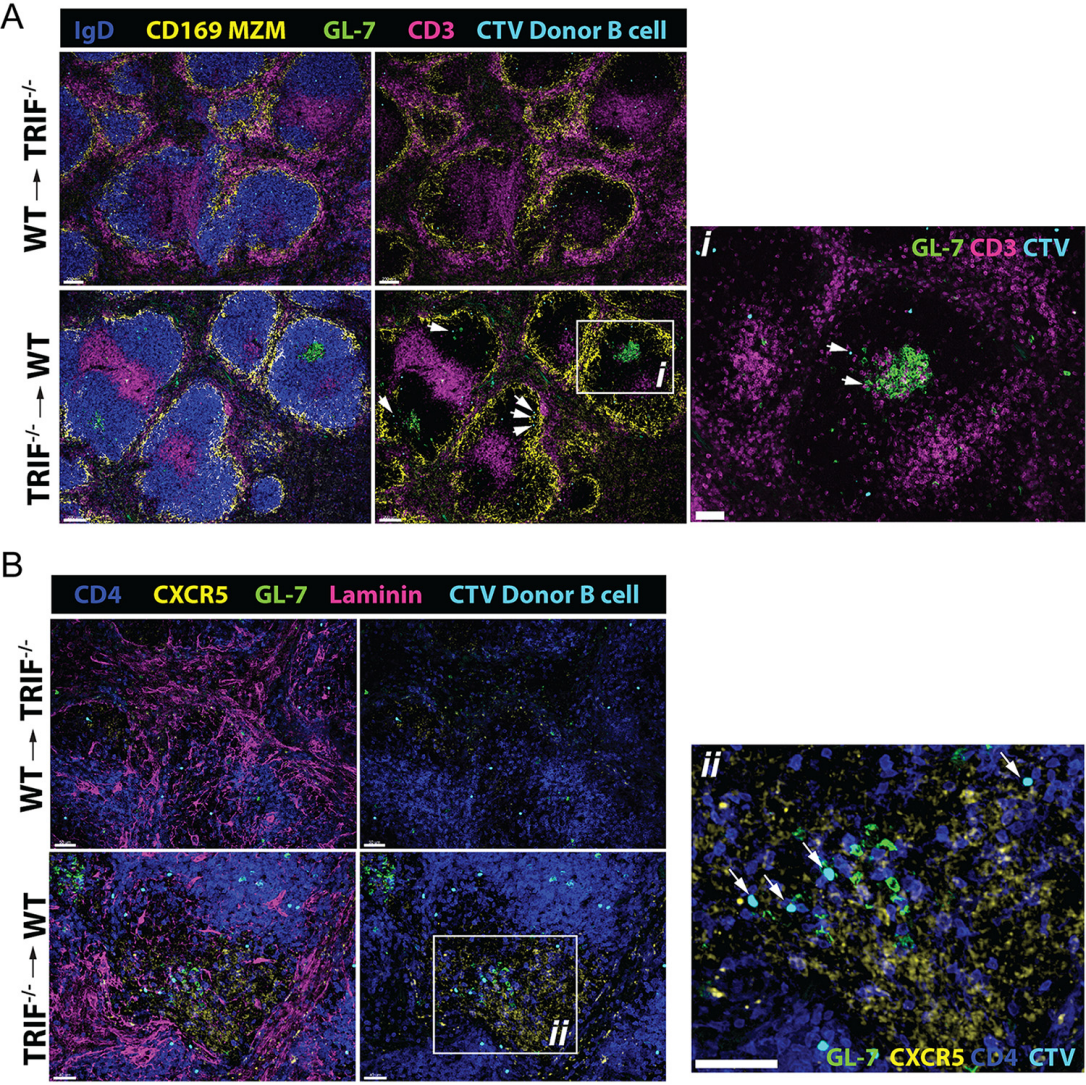
Immunofluorescence microscopy of early GC responses in the spleens of WT and TRIF^−/−^ mice after RRV infection. (A) Confocal micrograph of spleens from TRIF^−/−^ recipient mice that received CTV-labeled WT donor B cells (upper panels) and WT recipient mice that received CTV-labeled TRIF^−/−^ donor B cells (lower panels) 1 day prior to RRV infection. Images show spleen sections at 1 day post-RRV infection and donor CTV^+^ B cells in their respective mismatched recipients. Arrows in the lower panel and inset *i* show localization of donor B cells in proximity to recipient germinal centers. Sections were labeled for CD169^+^ marginal zone macrophages (MZM), IgD, CD3, and GL-7. Scale bar in inset *i**** ***=*** ***20 μm. (B) Confocal micrographs of spleen sections as described in panel A labeled for CXCR5^+^CD4^+^ T_FH_, laminin, and GL-7. Inset *ii* shows the region in the lower panel, and white arrows show donor CTV^+^ B cells in proximity to recipient T_FH_ and GC B cells. Scale bar in inset *ii**** ***=*** ***50 μm. Micrographs shown are representative of *n* = 5 mice per group.

Using confocal microscopy in the spleen, we observed that WT hosts that received TRIF^−/−^ B cells developed clear germinal centers, and donor B cells localized in close proximity to host GL-7^+^ cells, whereas this was not observed in TRIF^−/−^ hosts that received WT B cells (Fig. 7A and inset *i*). In addition, TRIF^−/−^ B cells colocalized in areas of the light zone replete with CXCR5^+^ CD4^+^ T cells in WT hosts, whereas this was not observed in TRIF^−/−^ hosts that received WT B cells (Fig. 7B and inset *ii*). This suggests that the inability of TRIF^−/−^ mice to efficiently undergo class switch recombination in the early stages of antiviral responses in RRV infection may be due to a lack of TRIF-dependent signaling within the local immune microenvironment rather than to an intrinsic defect in B cell function.

## DISCUSSION

This study investigated the role of TRIF in RRV disease using TRIF^−/−^ mice. TRIF^−/−^ mice infected with RRV experienced more rapid disease onset, indicating that TRIF may be essential for early-stage antiviral immunity. TRIF^−/−^ mice are more susceptible to HSV-1 and CHIKV infection than WT mice (20, 21, 30). The role of TRIF and TLR3 in RRV infection has not previously been reported, while TLR7 and Myd88 are recognized as potent mediators of the protective response against RRV (13). Our study is the first to show TRIF has an important role in IFN production during RRV infection and is important for an optimal immune response against RRV, with effects on B cell proliferation and T cells responses that impact antibody production and viral clearance.

Since TRIF is pivotal in type I IFN induction pathways, we examined production of type I IFN in RRV-infected TRIF^−/−^ mice. Compared to WT mice, type I IFN levels in serum and spleen were reduced on the first day following infection, confirming our hypothesis that TRIF-dependent signaling is a key contributor to type I IFN production. Our data also suggest that compensatory mechanisms for type I IFN induction may be at play in the absence of TRIF. Live virus titers in serum and spleen were higher in TRIF^−/−^ mice compared to WT mice at day 2 p.i., which is consistent not only with *in vitro* reports that TRIF-deficient human primary fibroblasts are more permissive to CHIKV replication (21), but also with *in vivo* studies showing that TRIF^−/−^ mice infected with CHIKV have higher viremia than WT mice (20). Our results therefore suggest that TRIF-dependent type I IFN induction is critical for control of RRV replication and viral clearance *in vivo.* Multiple host sensor pathways, including RIG-I, MDA5, IPS-1 (Cardif), TLR3, and MyD88, cooperate to control CHIKV infection (40). MyD88 acts as an adaptor for most TLRs, particularly TLR7 and TLR8, which both recognize viral single-stranded RNA in endosomes. TRIF, however, is the only adaptor for TLR3. Therefore, together with Her et al. (21), our report provides further evidence that TLR3 is a key contributor to type I IFN induction in alphavirus infection. The lower levels of type I IFN in TRIF^−/−^ mice were principally observed on day 1 postinfection, indicating the TLR3-TRIF axis is initiated early in alphavirus infection.

To further understand the immune response to RRV infection in a type I IFN-impaired background, we examined the expression profile of key cytokines implicated in RRV disease pathogenesis. IL-15 and CCL5 were significantly upregulated in the quadriceps of RRV-infected TRIF^−/−^ mice compared to WT mice. IL-15 is a potent regulator of T cells, promoting proliferation of memory CD8^+^ T cells, regulatory T cells (Treg cells), Th17 cells, and Th1/Th17 (IFN-γ- and IL-17-producing) cells (37). IL-15 also plays a central role in NK cell development by inducing differentiation of NK cells from their precursors (37, 38) and is required for formation and maintenance of memory CD8^+^ and CD4^+^ T cells (41–43). IL-15 is also essential for stimulating CD8^+^ T cell priming during HSV-1 infection (44). IL-15 plays an essential role in NK cell development and differentiation, since the absence of IL-15 or any of its receptor subunits results in profound NK cell deficiency (45). Therefore, the elevated levels of IL-15 and CCL5 in the RRV-infected TRIF ^−/−^ mice may account for the restoration of T cells and NK cells on day 7 p.i.

Histological analysis revealed no gross difference in cell infiltration in the muscles of RRV-infected TRIF^−/−^ mice compared to WT mice, although the numbers of CD4^+^ T cells, CD8^+^ T cells, and NK cells were all reduced in TRIF^−/−^ mice. CD69 can be induced by type I IFN (46) to act as an inhibitor of sphingosine-1-phosphate receptor 1 (S1P1) and is an early T cell activation marker that controls helper T cell differentiation (47, 48). Compared to WT mice, the proportion and total number of CD69^+^ CD4^+^ T cells, CD8^+^ T cells, and NK cells were significantly lower in TRIF^−/−^ mice at day 5 p.i. These cell numbers recovered to levels similar to those of WT mice by day 7 p.i. The number of CD4^+^ T cells was elevated in the quadriceps of RRV-infected WT mice, suggesting that CD4^+^ T cells are associated with RRV disease pathogenesis (49). Similar to our observations, the number of CD4^+^ T cells in the footpad of CHIKV-infected mice was reduced by half in TLR3^−/−^ mice compared to WT mice, indicating the CD4^+^ T cells play an essential role in disease pathogenesis by infiltrating the joint footpad and mediating inflammation in a TRIF and/or type I IFN-dependent manner (21). Thus, our observation of a reduced number of CD4^+^ T cells in the muscle tissue of RRV-infected TRIF^−/−^ mice is consistent with these previous reports. CD8^+^ T cell numbers are elevated in lymphoid and musculoskeletal tissues following RRV infection (50). Mice deficient in CD8^+^ T cells or depleted of CD8^+^ T cells showed increased RRV replication in skeletal muscle tissues, suggesting a potential role for CD8^+^ T cells in limiting alphavirus replication (50). In DENV infection, reports indicate an increase in the number of CD8^+^ T cells, and depletion of CD8^+^ T cells resulted in heightened viral loads, indicating that CD8^+^ T cells play a protective role in flavivirus infection (51–53). Likewise, NK cells have been reported in inflammatory infiltrates not only in animal models of RRV disease, but also in synovial exudates of patients infected with RRV, indicating an important role in the pathogenesis of alphavirus-induced arthritis (49, 54–59). Together with these reports, our data suggest the decreased numbers of CD4^+^ T cells, CD8^+^ T cells, and NK cells in TRIF^−/−^ mice at day 5 p.i. may partly account for the early onset of the disease symptoms observed in these mice. The restoration of CD4^+^ T cell, CD8^+^ T cell, and NK cell numbers to levels comparable to those of WT mice at day 7 p.i. may explain why TRIF^−/−^ mice display a peak disease profile similar to that of WT mice at days 10 to 12 p.i.

The type I IFN response is vital for protection from alphaviral infection and associated arthritic disease. Type I IFN promotes transcription of potent antiviral genes, including interferon-stimulated genes (ISGs). ISGs exert a plethora of effector functions that are critical in controlling replication of alphaviruses, including CHIKV, RRV, SINV, and O’nyong nyong virus (ONNV) infections (60–63). Some ISGs, such as protein kinase R (PKR), Mx GTPase, and RNase L, are critical to the induction of innate immune responses (64, 65). The type I IFN system also modulates adaptive immunity by promoting the activation of antigen-presenting dendritic cells and NK cells, thus impacting the localization, expansion, or differentiation of T cells and antibody-producing B cells (66, 67). Additionally, during viral infection, NK cell activation relies on type I IFN signaling (68–70), while NK cell activation by poly(I·C) occurs via a TRIF-dependent pathway (71). Therefore, the lower numbers of NK cells in TRIF^−/−^ mice at day 5 p.i. may be the result of impaired TRIF signaling and weakened type I IFN responses. Priming of both CD4^+^ and CD8^+^ T cells requires type I IFN signaling (10, 72, 73), and CD4^+^ and CD8^+^ T cell recruitment was shown to be impaired in type I IFN receptor α1 chain-deficient (CD118^−/−^) mice infected with HSV-1, pointing to a major role for type I IFN in the modulation of T cell priming during viral infection (74). Similarly, CD4^+^ T cell differentiation was shown to be driven by type I IFN in synergy with other cytokines, such as IL-18 and IL-21 (10). Thus, the reduced type I IFN levels in TRIF^−/−^ mice may lead to the impaired CD4^+^ T cell response. In contrast, CD8^+^ T cells may not be strictly dependent on type I IFN, as they have been shown to undergo activation in IFN-α/β receptor (IFNAR) knockout (KO) mice upon infection with a mouse-passaged DENV-2 strain (53). However, inhibition of type I IFN signaling at later time points using an anti-IFNAR neutralizing antibody led to dysfunctional CD8^+^ T cells in mice infected with West Nile virus (WNV), indicating that type I IFN may act as a redundant induction mechanism in the early phase of the CD8^+^ T cells response to arbovirus infection (75). Furthermore, in the absence of a functional type I IFN system, CD8^+^ T cell responses to acute lymphocytic choriomeningitis virus (LCMV) infection can instead rely on CD4^+^ T cell help (72). However, in TRIF^−/−^ mice, impaired CD8^+^ T cell responses appeared to correlate with curtailed type I IFN response in the early time points p.i., indicating an alternative mechanism for CD8^+^ T cell priming and expansion based on TRIF-dependent viral recognition and subsequent type I IFN induction.

The production of RRV-specific antibodies requires robust germinal center (GC) B cell formation. In our study, impaired GC formation was associated with lower titers of anti-RRV IgG in TRIF^−/−^ mice. Neighbours et al., showed that RRV neutralizing antibody and GC B cell activity were impaired in TLR7^−/−^ and Myd88^−/−^ mice infected with RRV, suggesting an essential role for the TLR7-MyD88 axis in B cell proliferation and differentiation (13). Our data show that TRIF is an important factor in the immune microenvironment that enables B cells to undergo early-stage differentiation and proliferation. As CD4^+^ T_FH_ cells play a stimulatory role in GC formation (76), reduced IgG titers and GC formation in RRV-infected TRIF^−/−^ mice may also be an indirect consequence of the impaired CD4^+^ T cell response, and therefore less efficient T_FH_-dependent stimulatory function, at early time points following RRV infection. This role for TRIF in T_FH_ differentiation and class switch recombination has previously been shown in E. coli infection (77), but to our knowledge, this is the first report of such a role in arthritogenic alphavirus infection.

RRV-specific antibodies are important for viral clearance and control of viral persistence. Transferring antisera from RRV-infected mice to naive mice prior to RRV infection led to a dramatic decrease in severity of RRV disease, suggesting an essential protective role for neutralizing antibodies. Antisera from RRV-infected TRIF^−/−^ mice provided a lower protection from *de novo* RRV infection, indicating TRIF plays an important role in driving the production of neutralizing antibody against RRV. Neutralizing IgG has been reported to suppress virus recrudescence and reduce viral antigen expression following mouse hepatitis virus (MHV) infection (78). Similarly, LCMV was shown to reactivate in mice lacking neutralizing antibodies (79). Our observation that RRV persists longer in TRIF^−/−^ mice where IgG antibody production is impaired is in line with these previous reports.

Collectively, our data show that TRIF^−/−^ mice are more susceptible to RRV infection and that type I IFN production was lower than in WT mice and was associated with higher viral replication—and persistence—in TRIF^−/−^ mice. The activation profile and abundance of CD4^+^ T cells, CD8^+^ T cells, and NK cells were all reduced in TRIF^−/−^ mice in the preacute phase of the disease. This may be a result of a curtailed type I IFN system, along with impaired class switch recombination likely resulting from poor GC B cell development in the absence of TRIF in the immune microenvironment. Thus, delayed type I IFN signaling coupled with an impaired IgG response is likely to hinder virus clearance and prolong disease in TRIF^−/−^ infected mice.

## MATERIALS AND METHODS

Information on the cells and viruses used is provided in the supplemental material. Standard virology, molecular biology, immunology, and *in vivo* methods used in this investigation are also described in the supplemental material.

### FACS sorting and B cell labeling.

B cells were isolated from pooled spleens and lymph nodes of WT and TRIF^−/−^ mice. Lymphoid tissues were mechanically disrupted in RPMI supplemented with 10% FCS and filtered through a 70-μm cell strainer. Cells were labeled using anti-mouse CD45 (30-F11), B220 (RA3-6B2), and non-B cells excluded using a dump gate (CD11b [(M1/70)], CD3 [(17A2)] and IA/IE [M5/114]). Dead cells were excluded using DRAQ7 (Biotium). Cells were sorted to 98.9% purity into FACS sorting buffer (Hanks balanced salt solution [HBSS] supplemented with 20% sterile FCS) on a BD LSR Aria II cell sorter with an 85-μm nozzle and kept in cold sorting buffer until labeling.

### Labeling of B cells and adoptive transfer.

Sorted B cells were counted using a hemocytometer and subsequently labeled using CellTrace violet (CTV; Thermo Fisher) proliferation dye according to the manufacturer’s instructions. Briefly, B cells were resuspended at 5 million cells/mL in sterile phosphate-buffered saline (PBS) and labeled with 1 μL of 5 mM CTV stock solution (final concentration of 5 μM) for 20 min at 37°C in the dark. The labeling reaction was quenched by adding a 5× volume of RPMI supplemented with 10% FCS, and cells were incubated for a further 5 min. Cells were centrifuged at 270 × *g* and resuspended in serum-free HBSS. Then, 2.5 × 10^5^ CTV-labeled B cells were adoptively transferred intravenously into mismatched 21-day-old recipient mice (e.g., TRIF^−/−^ CTV^+^ B cells into WT recipients and WT CTV^+^ B cells into TRIF^−/−^ recipients) 1 day prior to infection with RRV.

### Statistical analysis.

Mouse clinical scores, cytokine expression qRT-PCR, antibody ELISA, type I IFN ELISA, cell infiltrates for H&E staining, flow cytometry assay, and plaque assays were analyzed using two-way analysis of variance (ANOVA) with a Bonferroni *post hoc* test. Antibody ELISA (day 10 p.i.), antibody neutralizing assay, qPCR for viral positive/negative genome RNA, and ISG54 were analyzed using Student’s *t* test. All data (except for mouse clinical scores) were tested for normality using the D’Agostino-Pearson normality test prior to analysis. All statistical analyses were performed with GraphPad Prism software version 7.

### Data availability.

All relevant data are within the paper and its supporting information file.

10.1128/mbio.03363-21.1 TEXT S1 Supplemental methods. Download Text S1, DOCX file, 0.07 MB. Copyright © 2022 Liu et al. 2022 Liu et al. https://creativecommons.org/licenses/by/4.0/ This content is distributed under the terms of the Creative Commons Attribution 4.0 International license.
