# Supplementary material for: TIR-Domain-Containing Adapter-Inducing Interferon-β (TRIF)-Dependent Antiviral Responses Protect Mice against Ross River Virus Disease
Source: mBio. 2022 Jan 4;13(1):e03363-21. doi: 10.1128/mbio.03363-21 (PMC8725586; doi:10.1128/mbio.03363-21)
Supplement: TEXT S1 [file mbio.03363-21-s0001.docx]

**Supplemental Methods**

**Ethics statement**

All animal experiments were approved by the Animal Ethics Committee of Griffith University (GLY/03/14/AEC). All procedures conformed to the National Health and Medical Research Council (80).

**Virus**

The prototype Ross River virus T48 (kindly provided by Dr. Richard Kuhn, Purdue University) was originally isolated from *Aedes vigilax* in northeastern Queensland and passaged 10 times in suckling mice and twice in Vero cells prior to generating an infectious clone (81, 82). The RRV-T48 virus stock was generated by *in vitro* transcription (mMESSAGE mMACHINE SP6 Transcription Kit, Ambion) as previously described (81). Briefly, 2 μg of RRV-T48 cDNA plasmids were linearized, transcribed and electroporated into Vero cells using an electroporator (Eppendorf). Recovered viruses were amplified in Vero cells. The virus titers were determined by standard plaque forming assay on Vero cells (83). The virus stocks were stored in 0.5 ml aliquots at −80 °C.

**Mouse infections and disease monitoring**

C57BL/6 WT mice were originally obtained from the Animal Resources Centre (Perth, Australia). The C57BL/6 TRIF^-/-^ mice (84) were kindly donated by Dr Shizuo Akira (Osaka University) and colonies bred and maintained at QIMR Berghofer (Brisbane, Australia). Twenty-day old C57BL/6 WT and TRIF^-/-^ mice were inoculated subcutaneously in the thorax below the right fore limb with 10^4^ PFU of RRV-T48 diluted in PBS to a volume of 50 μl (85). Mock-infected mice were inoculated with PBS only. Mice were weighed and scored for disease daily. RRV disease scores were assessed based on strength and hind-leg dysfunction using the following scale: 0, no disease signs; 1, ruffled fur; 2, very mild hind limb weakness; 3, mild hind limb weakness; 4, moderate hind limb weakness and dragging of hind limbs; 5, severe hind limb weakness/dragging; 6, complete loss of hind limb function; 7, moribund. Humane end point was defined as mice scoring 7 in clinical disease or showing weight loss of greater than 15% of their starting weight (49).

**Plaque assay**

Vero cell monolayers were infected with RRV test samples prior to the addition of 0.2% agarose overlay. Plates were incubated for an additional 48 h, after which cells were fixed with 4% formaldehyde and 0.05% crystal violet. Viral titers were calculated using the following formula: PFU/ml = [Average plaques/Volume (ml) of virus added] × dilution factor. (54).

**PRNT assay**

RRV neutralizing antibody levels were determined by plaque reduction neutralization test (PRNT) assay. Briefly, RRV T48 infected mice were culled and their serum was collected. Serum was heat inactivated at 56 °C for 30 min and then underwent serial 2-fold dilutions. The serum samples were combined with RRV T48 (10^3^ PFU) and incubated at 37 °C for 30 min. The viral titer of these serum-virus mixtures were determined by plaque assay. The levels of the RRV neutralizing antibody in mouse serum were expressed as the reciprocal of the fold-dilution which reduced 50% of the original RRV T48 titer.

**H&E histological analysis**

RRV-T48 infected mice were culled, and the quadriceps were collected and fixed in 4% paraformaldehyde (PFA), followed by paraffin embedding. Samples were cut into 5 μm thick sections and stained with H&E. Images were taken using Nikon Eclipse TS100 inverted microscope. Tissue cellular infiltrates were quantified using ImageScope software (Algorithm Nuclear v9) using the following threshold settings: Min Nuclear Size (µm^2^) = 20; Max Nuclear Size (µm^2^) = 200; Min Roundness = 0.4; Min Compactness = 0.4; Min Elongation = 0.2. Analysis of histology was done in a blinded fashion by two independent observers.

**Flow cytometry**

RRV-T48 infected mice were culled and perfused with PBS. Spleens and quadriceps were collected for FACS analysis. Quadriceps were minced and digested in Type IV Collagenase (3 mg/ml; Worthington) with DNase I (10 mg/mL; Sigma) and passed through 70 and 30 µm cell strainers. Spleens were manually homogenized and passed through 70 and 30 µm cell strainers. Cells were stained with antibodies for 45 min in FACS buffer (PBS with 2% FBS v/v and 0.5 mM EDTA). Fluorochrome-conjugated monoclonal antibodies against mouse CD45 (30-F11), CD3 (17A2), CD8 (53.6-7), CD4 (RM.4-5), CD69 (H1.2F3), NK1.1 (PK136), Ly6C (HK1.4), CD11b (M1/70), IgD (11-26c (11-26)), GL-7 (14-5902-82), B220 (RA3-6B2) and CXCR5 (2G8) (all purchased from eBiosciences) were used, and NIR (near infra-red) LIVE/DEAD stain (Thermo Fisher) was used to exclude dead cells. Counting beads (Spherobeads, BD) were added to the samples before acquisition. Cell populations were analyzed on a BD LSRFortessa cell analyzer with BD FACSDiva software, version 6.1.3. Data analysis was performed with FlowJo (TreeStar, Inc.) software, version 9.0.

**ELISA**

RRV-T48 infected mice were tail-bled to obtain serum samples. 96-well Nunc MaxiSorp ELISA Plates were coated with purified RRV-T48 at 20000 PFU/ml overnight and then blocked with blocking buffer for 2 h. 50 μl diluted serum samples were added to each well of the plates and incubated for 1 h. The plates were washed with washing buffer and then goat anti-mouse IgM µ chain (HRP) (Abcam) or anti-mouse IgG, HRP-linked antibody (Cell signaling) was added at a dilution of 1:5000. After incubation for 1 h, the antibodies were removed and the plates were washed with washing buffer. TMB substrate (Thermo Fisher) was added to the wells and the reactions were stopped with H_2_SO_4_. The plates were read in an ELISA plate reader at 450 nm wavelength.

The levels of IFN-β in the mouse serum were determined by LEGEND MAX Mouse IFN-β ELISA Kit (BioLegend, Cat# 439407) according to the manufacturer’s instructions.

**Immunofluorescent histology and confocal microscopy.**

RRV-T48 infected mice were culled and perfused with PBS. Foot-draining popliteal lymph nodes and spleens were collected, fixed in 4% PFA, dehydrated in 30% sucrose (w/v in PBS) and cryoembedded in OCT prior to sectioning using a Leica CM1850UV cryostat. Lymph nodes (LN) were cryosectioned into 14 μm-thick sections. LN cryosections were immunolabelled using rat anti-mouse MHC-II (BD Biosciences), rat anti-mouse GL-7-AlexaFluor 488 (Biolegend), rat anti-mouse B220-Pacific Blue (BD Biosciences) and rabbit anti-mouse collagen IV (AbD Serotec). Primary antibodies were detected using anti-rat and anti-rabbit secondary antibodies conjugated to AlexaFluor 647 and AlexaFluor 568. Spleen cryosections were cut at 30 μm thickness and rehydrated with 0.5% bovine serum albumin (BSA) in PBS for 20 minutes. Sections were subsequently blocked using Serum-Free Protein Block solution (DAKO) for 45 minutes at room temperature. Where applicable, endogenous biotin was quenched using a Biotin-Avidin blocking kit (DAKO). Sections were immunolabelled using rat anti-mouse CD169 (AbD Serotec), biotinylated rat anti-mouse CXCR5 (BD Biosciences), rabbit anti-mouse laminin (AbD Serotec), streptavidin-conjugated DyLight 570 (eBiosciences), rat anti-mouse IgD-AlexaFluor 647, rat anti-mouse CD4-AlexaFluor 647, rat anti-GL-7-AlexaFluor 488, rat ant-mouse CD3-AlexaFluor 700 (all from BD Biosciences). Tissue sections were mounted using ProLong Gold Antifade and z-stacks were acquired using an Olympus FV3000 confocal microscope and processed as a maximum intensity projection (MIP). Raw images were processed using Imaris 9.5 software (Bitplane) and where applicable, spectral spillover was corrected using the Imaris XT plugin Channel Arithmetics.

**Qualitative Real-Time PCR (q-PCR) assay for mouse cytokines**

RRV-T48 infected mice were culled and perfused with PBS. The quadriceps were collected at day 5 and 7 p.i. Total RNA was extracted using TRIzol (Invitrogen) according to the manufacturer’s instructions. The RNA was reverse transcribed into cDNA using reverse transcriptase (Sigma-Aldrich) with random primers. SYBR Green Real-Time PCR was performed on a CFX96 touch Real-Time PCR system (Bio-rad). The PCR conditions were set as: (1) 95 °C for 15 min, 1 cycle; (2) 94 °C for 15 s, followed by 55 °C for 30 s and 72 °C for 30 s, 40 cycles. The DNA amplification specificity was evaluated by melting curve analysis. The mRNA level of each target gene was expressed as fold change relative to mock infected samples (86).

**qRT-PCR assay for viral positive strand and negative strand RNA**

RRV-T48 infected mice were culled and perfused with PBS. The quadriceps were collected at day 18 p.i. The total RNA was extracted using TRIzol (Invitrogen) according to the manufacturer’s instructions. For RRV positive strand RNA, the total RNA was reverse transcribed into cDNA using reverse transcriptase (Sigma-Aldrich) with random primers. QuantiTect® Probe Real-Time PCR was performed on a CFX96 touch Real-Time PCR system (Bio-rad). RRV-T48 nSP3-targeting Primer sequences: Forward 5’ CCGTGGCGGGTATTATCAAT 3’ and Reverse 5’ AACACTCCCGTCGACAACAGA 3’. Probe sequence (6-FAM)-ATTAAGAGTGTAGCCATCC-(TAMRA) (87). The PCR conditions were set as: (1) 95 °C for 15 min, 1 cycle; (2) 94 °C for 15 s, followed by 60 °C for 1 min, 40 cycles. The DNA amplification specificity was evaluated by melting curve analysis. A viral infectious clone DNA standard curve was generated and based on which the viral genome RNA was calculated. For RRV negative strand RNA, the total RNA was reverse transcribed into cDNA using primer 5’ GGCAGTATCGTGAATTCGATGCGATCTGGAATCTCCAACGTT 3’. SYBR Green Real-Time PCR was performed on a CFX96 touch Real-Time PCR system (Bio-rad) using primers: Forward 5’ AATAAATCATAAGGCAGTATCGTGAATTCGATGC 3’ and Reverse 5’ AATAAATCATAATTCAGCTCTAGTGCGACACGT 3’. The PCR conditions were set as: (1) 95 °C for 15 min, 1 cycle; (2) 94 °C for 15 s, followed by 55 °C for 30 s and 72 °C for 30 s, 40 cycles. The DNA amplification specificity was evaluated by melting curve analysis. A viral infectious clone DNA standard curve was generated and based on which the viral negative strand RNA was calculated.

**Passive serum transfer**

Twenty-day old C57BL/6 WT and TRIF^-/-^ mice were inoculated subcutaneously with 10^4^ PFU of RRV-T48. The infected mice were culled at day 10 p.i. and the sera were collected and pooled together according to strain. The sera were heat inactivated at 56 °C for one hour and stored at -80 ºC until use. Twenty-day old C57BL/6 WT and TRIF^-/-^ mice were inoculated intraperitoneally with 50 µl of the heat inactivated WT or TRIF^-/-^ antisera and infected with 10^4^ PFU RRV-T48 subcutaneously at one hour after the serum transfer (13). The mice were monitored daily for disease signs as described above.
